# A Randomized Study Evaluating Clinical Efficacy and Safety of Trusteel® and Ethisteel® Surgical Steel Sutures for Sternal Closure in Subjects Undergoing Surgical Procedures by Sternotomy

**DOI:** 10.7759/cureus.58715

**Published:** 2024-04-22

**Authors:** G Rama Subrahmanyam, Ramji Mehrotra, N. L. Sailaja Vasireddy, Abdus Samad, Ashok K Moharana, Deepak Siddabasavaiah

**Affiliations:** 1 Cardio-Thoracic Surgery, Care Hospital, Hyderabad, IND; 2 Cardio-Thoracic & Vascular Surgery, BLK-Max Super Speciality Hospital, New Delhi, IND; 3 Clinical Affairs, Healthium Medtech Limited, Bengaluru, IND

**Keywords:** wound pain, sternotomy, sternal dehiscence, stainless steel suture, quality of life

## Abstract

Introduction: Sternal dehiscence and other post-sternotomy complications, viz. superficial and deep sternal wound infection, mediastinitis, and sternal instability increase the risk of mortality. Sternotomy closure with steel sutures results in a low complication rate. Therefore, this study compared the clinical equivalence of Trusteel^®^ (Healthium Medtech Limited, Bengaluru, India) and Ethisteel^®^ (Ethicon, Johnson & Johnson, Cincinnati, USA) surgical steel sutures for sternal closure following median sternotomy.

Methods: The primary endpoint of this prospective, single-blind, multicentric, two-arm, randomized (1:1) study (April 2021-April 2023) was a comparison of the proportion of subjects having sternal dehiscence within 26 weeks of the median sternotomy closure between Trusteel^®^ (n=33) and Ethisteel^®^ (n=34) groups. Secondary endpoints comprised an assessment of intraoperative suture handling, the incidence of mortality and other complications of sternal closure, operative time, intensive care unit (ICU)/hospital stay, return to normal day-to-day activities and work, subject satisfaction and general well-being, and adverse events in both groups. A statistically significant result between the groups was considered at p<0.05.

Results: No incidence of sternal dehiscence or other post-operative complications were recorded. A significant difference (p<0.05) in the stretch capacity of Trusteel^®^ and Ethisteel^®^ sutures was noted; otherwise, ease of passage, knot holding, knot security, knot tie-down smoothness, and memory of both sutures had comparable ratings. Operative time, ICU/hospital stay, and return to normal day-to-day activities and work were comparable between the groups. Improvement in post-operative functional abilities, quality of life, and health status was evident in both groups and was comparable.

Conclusion: Trusteel^®^ surgical steel suture is clinically equivalent to Ethisteel^®^ surgical steel suture and is safe and effective for sternal closure following median sternotomy.

## Introduction

Since the 1950s, cardiac surgery through median sternotomy has been considered the best clinical incision of choice for subjects with multiple vessel disease and co-morbidities [1]. Although one million surgeries have been performed globally in the last two decades, the occurrence rate of sternal complications remains unchanged [2,3]. Sternal split is considered the major risk factor for the development of wound complications, such as dehiscence and infection [4]. Sternal instability or dehiscence can be categorized as mechanically uninfected, superficial and deep sternal wound infection, or mediastinitis [5]. Mediastinitis with sternal dehiscence is reported to cause potentially fatal complications resulting in 14-47% mortality cases [2,5]. Deep sternal wound infection is associated with increased medical costs, prolonged hospitalization as well as reoperation [2]. Therefore, establishing an effective method of sternal closure that results in minimal sternal wound complication and assists in recovery and mobility is particularly necessary [6].

Sternal integrity resumption following sternotomy for open heart surgery forms the basis for speedy recovery [4]. Sternotomy closure with wires has been the gold standard technique for more than 50 years as it is easily and quickly achievable, has a low complication rate, and is economical because of the low cost of steel wires [7]. Presently, there is a paucity of evidence regarding the comparative outcomes of two commonly used brands of monofilament stainless steel sutures for sternum closure. The present study was designed to compare the clinical equivalence of Trusteel® (Healthium Medtech Limited, Bengaluru, India) and Ethisteel® (Ethicon, Johnson & Johnson, Cincinnati, USA) stainless steel suture for closure of the sternum in subjects undergoing procedures by median sternotomy.

## Materials and methods

Study design

A multicentric, prospective, two-arm, parallel-group, randomized, single-blind study was conducted between April 2021 and April 2023 in the Cardio-Thoracic department of two tertiary care centers in India (i) Care Hospital, Hyderabad and (ii) BLK-Max Super Speciality Hospital, New Delhi. The primary study objective was to compare the rate of sternal dehiscence after median sternotomy closure between Trusteel® and Ethisteel® suture groups. The secondary objectives of this study were evaluation of all-cause mortality, other surgical complications of median sternotomy closure, overall intraoperative handling of suture, time taken to return to work and normal day-to-day activities, adverse events (AEs), and overall subject satisfaction score and well-being in the two groups.

Ethical approval

The trial registration is under the Clinical Trial Registry of India (registration number: CTRI/2020/10/028240; registered on 05/10/2020). Approval of the ethics committee from all the clinical study sites was acquired prior to the initiation of the trial. In addition, the declaration of Helsinki, ICH-GCP E6 R2, EN ISO 14155:2020, MDR (EU) 2017/745, Indian MDR rules 2017, New Drugs and CT rules 2019, and Consolidated Standards of Reporting Trials (CONSORT) guidelines were followed.

Study participants

The study included adults (18-70 years) who were scheduled for median sternotomy for surgical procedures related to heart, great vessels, or mediastinal lesions after obtaining informed consent.

Participants with a history of median sternotomy, allergy to steel or similar products, or bleeding disorders were excluded. Participants, who were pregnant or used the experimental drug or medical device within 30 days prior to the present study or were already participating in another cardiovascular or similar study, or employees of the investigator or study center with direct involvement in the proposed study or other studies under the direction of that investigator or study center, were excluded. Participants with the American Society of Anesthesiologists (ASA) classification V, active infection at/around the skin incision site, mental disorder, learning disability or language barrier, or any other indication-based exclusion in the opinion of the investigator that might result in non-compliance with the surgical procedure or completion of the study were also excluded.

Intervention

Trusteel® (Healthium Medtech Limited) and Ethisteel® (Ethicon, Johnson & Johnson) are non-absorbable surgical sutures, composed of 316LVM and 316L stainless steel respectively. Both sutures are indicated for use in abdominal wound closure, sternal closure, and orthopedic procedures including cerclage and tendon repair.

Study procedure

Median sternotomy was performed conventionally. All routine aseptic precautions were taken pre-operatively, during the surgery, and post-operatively following the existing standards. After completion of the surgical procedures and achievement of hemostasis, either Trusteel® or Ethisteel® stainless steel monofilament wire suture was placed to coapt the sternum at multiple points from the manubrium sterni to the most caudal portion of the corpus sterni. The standard institutional protocol was followed to close the pre-sternal fascia, muscle, and skin. A sterile wound dressing was done.

The subjects were screened from Week -26 to Day -1 and underwent surgery on Day 0 (baseline). The study outcomes were investigated for the next 26 weeks on the scheduled post-operative follow-ups: Day 3, Day 4-15, i.e., day of discharge (DOD), Week 6 post-DOD, Week 12 post-DOD, and Week 24 post-DOD.

Demographic and other relevant characteristics

Baseline demographics (age, ethnicity, gender, height, weight, body mass index, occupation, ASA classification, and smoking and alcohol history), family history of cardiovascular disease, reason for sternotomy, medical or surgery history, and physical examination for any problem were evaluated.

Study outcomes

Primary Endpoints

Primary endpoints, a comparison of the proportion of subjects having sternal dehiscence within 26 weeks of the median sternotomy closure, were assessed between the groups on all post-operative follow-ups. The diagnosis was made based on clinical findings of a sternal click or evidence of sternal instability during coughing or respiration.

Secondary Endpoints

Intraoperative suture handling characteristics in terms of ease of passage through tissue, first-throw knot holding, knot tie-down smoothness, knot security, stretch capacity, and memory were rated by the investigator as “Poor” (scale 1), “Fair” (scale 2), “Good” (scale 3), “Very good” (scale 4) and “Excellent” (scale 5). Details of antibiotic and thrombosis prophylaxis, operative time (from skin incision to the end of skin closure), technique of sternotomy closure, sternotomy incision length, size and number of sutures used, blood transfusion, perioperative complications (if any), and outcome of surgery were noted.

At baseline (after the surgery) and at all post-operative follow-ups, the incidence of mortality, related both to native disease and wound problems, and post-operative suture-related challenges were recorded. Other complications of sternal closure, such as superficial, and deep sternal wound infection, mediastinitis, reoperation rate due to sternal dehiscence, and wire fracture on all post-operative follow-ups were noted. Sternal instability was evaluated on a 4-point scale, where 0 indicated “clinically stable normal sternum with no detectable motion” and 4 indicated “completely separated sternum”. A chest radiograph was done based on the investigator's discretion and institutional protocol to evaluate the mid-sternum line of lucency, sternum wire displacement, and obvious interruption/dislocation. Subjective assessment of post-operative wound pain at rest, during coughing, and on movement was self-completed by the respondent using a visual analog scale (VAS) on Day 3, DOD, and at Week 6, 12, and 24 post-DOD. Pain was subjectively rated as “No pain” (score 0-4), “Mild” (score 5-44), “Moderate” (score 45-74), and “Severe” (score 75-100). In addition, a subjective measure of pain after recovery from anesthesia was performed using VAS. The frequency of coughing was noted on all post-operative follow-ups. The mean duration of days of stay in the intensive care unit (ICU) and the hospital, as well as the time taken to return to normal day-to-day activities and work, was recorded. Subject satisfaction and general well-being were assessed subjectively by the EuroQoL five-dimensional three levels (EQ-5D-3L) questionnaire. Five dimensions of the subject’s quality of life, viz. mobility, self-care, usual activities, pain/discomfort, and anxiety/depression were graded as “No problems”, “Some problems” and “Extreme problems”. EuroQol visual analogue scale (EQ-VAS) was used for the subjective rating of the subject’s health in values between 0 (worst imaginable health) and 100 (best imaginable health). Both EQ-5D-3L and EQ-VAS were measured at screening visits, DOD, and all post-DOD visits.

Any untoward medical occurrence during the study period whether had a causal relationship with the surgery/intervention or not, was recorded as an AE. If the unfavorable condition resulted in death or life-threatening circumstances requiring hospitalization, then it was captured as a serious adverse event (SAE).

Sample size

The rate of sternal dehiscence with steel wire closure (n=1336) was recorded as 0.3% by a previous study (Sharma et al., 2004) [8], which was taken into consideration while estimating the sample size of the present study. The Ethisteel® suture arm was assumed to have 0.3% subjects with sternal dehiscence. The anticipated cumulative proportion of the sternal dehiscence in the Trusteel® suture arm was assumed to be 0.6%. Considering 5% as type I error, 80% power, and 5% margin of non-inferiority, the sample size requirement was calculated as 28 subjects in each arm with a minimum total sample size requirement of 56 subjects. Furthermore, 30% dropout and post-randomization exclusion were considered for increasing the required sample size to 72 (n=36 in each arm). The sample size was calculated as follows:

Two- sample parallel - Non-inferiority - π_1_-π_2 _> δ - n_i_= (Z_α_ + Z_β_)^2^ (π_1 _(1-π_2_) + π_2_ (1-π_2_)) / (π1-π2-δ )^2^

n_i_: sample size required in each group; Z_α_: conventional multiplier for alpha; Z_β_: conventional multiplier for power; π_1_: sternal dehiscence in the standard Ethisteel® arm; π_2_: sternal dehiscence in Trusteel® arm; δ: margin of non-inferiority difference; and π_1_-π_2_: size of difference of clinical importance.

Randomization and blinding

Random Allocation Software, version 1.0 was used to generate two random lists (n=36) using block sizes of 4, 6, or 8. The generated numbers were masked by applying a sequentially numbered opaque sealed envelope technique. Eligible subjects were randomized in a 1:1 ratio to receive either of the two sutures and were unaware of the study intervention. The operating staff were not blinded.

Statistical analysis

Comparison between subjects of two suture arms, who had complete data on primary effectiveness parameters until 26 weeks follow-up, was performed without any major protocol deviations based on per-protocol or PP analysis set using IBM SPSS Statistics for Windows, Version 28 (Released 2021; IBM Corp., Armonk, New York, United States). The chi-square test was used for the comparison of qualitative data (proportions or percentages) of study outcomes including data on primary endpoint, and proportion of subjects having sternal dehiscence. All continuous data of parametric and non-parametric distribution were presented as mean±SD and compared using the t-test and Mann-Whitney U test, respectively. Data of secondary endpoints were compared as per the quantitative or qualitative nature of the variables. A statistically significant test result between the groups was considered at p<0.05.

## Results

**Figure 1:**
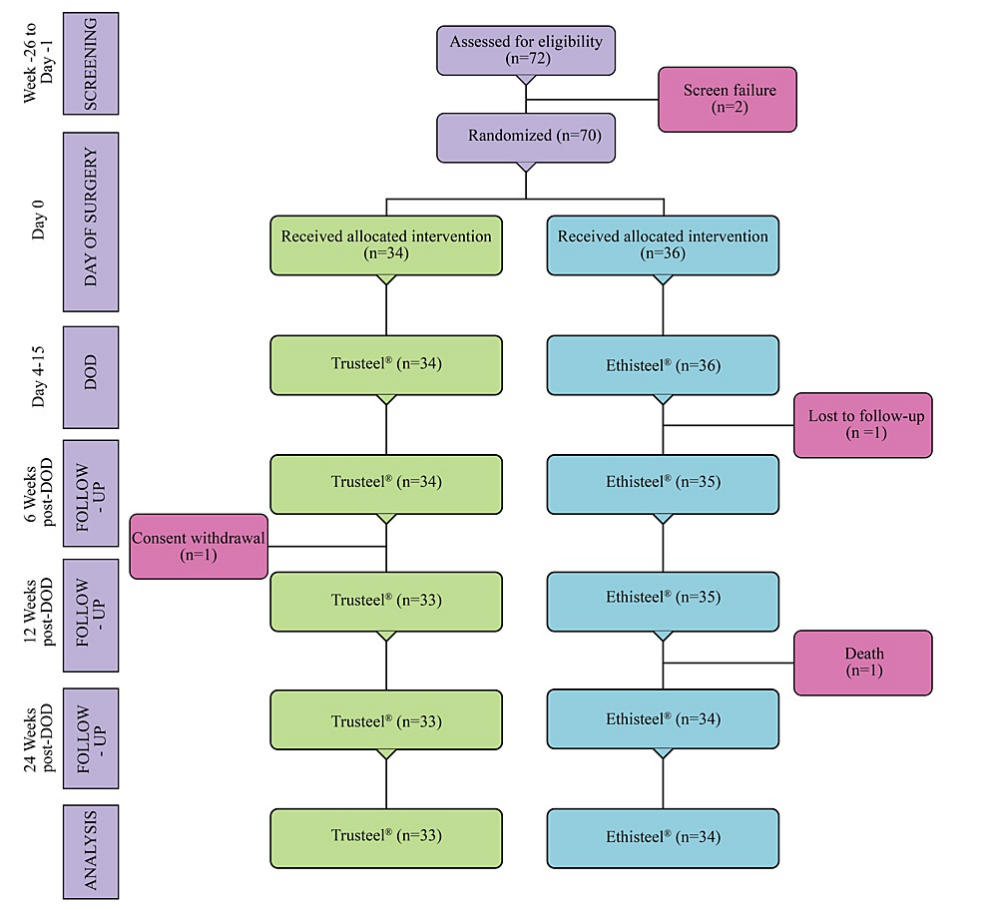
CONSORT diagram of study participants DOD: Day of discharge, n: Number of subjects CONSORT: Consolidated Standards of Reporting Trials Trusteel®: Healthium Medtech Limited, Bengaluru; Ethisteel®: Ethicon, Johnson & Johnson, Cincinnati, USA

The study was conducted with the screening of 72 eligible participants between April 2021 and October 2022. Two were screen failed, one was excluded owing to consent withdrawal, one was lost to follow-up, and one died due to index disease condition. A total of 67 subjects (Trusteel® (n=33) and Ethisteel® (n=34)) requiring sternotomy for open surgical procedures on the heart completed the study between April 2021 and April 2023 (Figure 1).

Out of 67 subjects, 31 (Trusteel®, n=16 and Ethisteel®, n=15) were randomized by one participating site and 36 (Trusteel®, n=17 and Ethisteel®, n=19) by another site.

Demographic and other relevant characteristics

All study participants were Indian, except one (3.0%) in the Trusteel® group, who was non-Indian Asian (p=0.31). The median subject age was 52.8 and 60.3 years in the Trusteel® and Ethisteel® groups, respectively. Occupation status of Trusteel® and Ethisteel® groups revealed that the majority had mild strenuous (63.6 vs. 58.8%) or desk (18.2 vs. 26.5%) jobs (p=0.70). Baseline demographics, family history of cardiovascular disease, and medical/surgical history were found to be comparable between the groups (Table 1).

**Table 1:** Baseline characteristics of study participants Data is presented as mean±SD or proportions (percentages) as applicable; t-test was used to compare all continuous data; ASA: American Society of Anesthesiologists, BMI: Body mass index; CKD: Chronic kidney disease; Body mass index ≥30.0 kg/m^2^ is obesity as per Centers for Disease Control and Prevention, kg: Kilogram, cm: Centimeter, kg/m^2^: kilogram per square meter, n: number of subjects Trusteel®: Healthium Medtech Limited, Bengaluru; Ethisteel®: Ethicon, Johnson & Johnson, Cincinnati, USA

Baseline characteristics	Trusteel^®^ (n=33)	Ethisteel^®^ (n=34)	p-value
Male	27 (81.8)	30 (88.2)	0.46
Age (years)	54.5 ± 7.9	56.9 ± 10.0	0.39
Weight (kg)	68.6 ± 12.0	69.5 ± 9.1	0.19
Height (cm)	163.5 ± 6.8	165.6 ± 8.9	0.20
BMI (kg/m^2^)	25.6 ± 4.00	25.4±2.7	0.18
Smoking history	4 (12.1)	4 (11.8)	0.96
Alcohol consumption history	3 (9.1)	3 (8.8)	0.97
Medical/surgical history	33 (100.0)	34 (100.0)	1.00
Comorbidities			
Obesity	2 (6.1)	2 (5.9)	
Diabetes	19 (57.6)	16 (47.1)	
CKD	1 (3.0)	0	0.98
ASA I	4 (12.1)	5 (14.7)	0.92
ASA II	16 (48.5)	15 (44.1)	
ASA III	13 (39.4)	14 (41.2)	
Family history of cardiovascular disease	1 (3.0)	0	0.31

In Trusteel® and Ethisteel® groups, 57.6% and 47.1% of subjects respectively had a history of diabetes. Physical examination documented abnormal cardiovascular system in all subjects (p=1.00).

Primary endpoint analysis

The primary endpoint, incidence of sternal dehiscence, was not reported in any subject of either Trusteel® and Ethisteel® group within 26 weeks of the median sternotomy closure (p=1.00).

Secondary endpoint analysis

Intraoperative Profile

Coronary artery bypass graft (CABG) surgery was the common procedure, performed in subjects of Trusteel® (87.9%) and Ethisteel® (97.1%) group. In the Trusteel® group, valve replacement surgery (9.1%) and atrial septal defect closure combined with CABG surgery (3.0%) were also done. Only one (2.9%) subject in the Ethisteel® group had aortic surgery. Suture size 6 of both Trusteel® and Ethisteel® sutures was used in 48.5% and 44.1% of subjects respectively, and suture size 7 of both Trusteel® and Ethisteel® sutures was used in 51.5% and 55.9% subjects respectively (p=0.72). The figure-of-8 configuration technique of sternum closure was used in the majority of subjects (54.5 vs. 52.9%), followed by the conventional technique (45.5 vs. 47.1%) in Trusteel® vs. Ethisteel® group (p=0.90). Sternotomy incision length, length of surgery, and number of blood transfusions were comparable between the groups (Table 2).

**Table 2:** Intraoperative and post-operative characteristics of study participants Data is presented as mean±SD; ^ t-test was used, #Mann-Whitney U test was used; ICU: Intensive care unit, cm: centimeter, n: number of subjects Trusteel®: Healthium Medtech Limited, Bengaluru; Ethisteel®: Ethicon, Johnson & Johnson, Cincinnati, USA

Subject profile	Trusteel^® ^(n=33)	Ethisteel^® ^(n=34)	p-value
Intraoperative			
Sternotomy incision length (cm)	15.6 ± 4.1	15.3 ± 4.2	0.73^^^
Length of surgery (hours)	3.5 ± 1.6	3.3 ± 1.5	0.88^#^
Number of blood transfusions	1.1 ± 0.7	1.2 ± 0. 9	0.65^#^
Post-operative			
Length of ICU stay (days)	4.6 ± 1.3	4.9 ± 1.2	0.49^^^
Length of hospital stay (days)	7.6 ± 0.8	7.9 ± 1.4	0.29^^^
Time taken to return to normal day-to-day activities (days)	10.2 ± 3.1	10.3 ± 3.2	0.31^^^
Time taken to return to work (days)	53.6 ± 46.7	56.1 ± 43.7	0.69^#^

**Figure 2:**
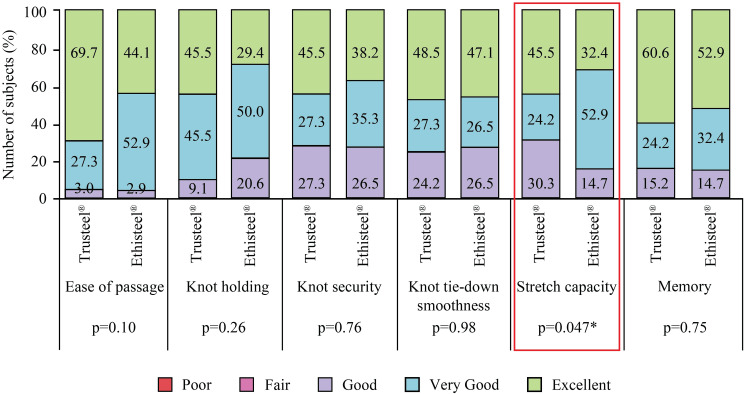
Intraoperative handling characteristics of Trusteel® (n=33) and Ethisteel® (n=34) sutures *p<0.05 Trusteel®: Healthium Medtech Limited, Bengaluru; Ethisteel®: Ethicon, Johnson & Johnson, Cincinnati, USA

Only one blood transfusion (69.7 vs. 76.5%) was required in the majority of subjects of the Trusteel® and Ethisteel® groups; four transfusions were used in only 5.9% of subjects of the Ethisteel® group (p=0.96). A minimum of three and a maximum of five sutures (median four sutures) were used in both groups. Three sutures were used in 9.1 and 26.5% of subjects, four were used in 78.8 and 70.6% of subjects, and five were used in 12.1 and 2.9% of subjects of Trusteel® and Ethisteel® groups (p=0.36). During the surgery, the investigators have encountered no suture-related challenges. Subjective evaluation of intraoperative handling showed “Excellent”, “Very good” or “Good” ease of passage, knot holding, knot security, knot tie-down smoothness, stretch capacity, and memory of both Trusteel® and Ethisteel® stainless steel sutures. A significant difference (p<0.05) was only recorded for the stretch capacity of Trusteel® and Ethisteel® sutures (Figure 2).

Cefuroxime was the most commonly used intraoperative antibiotic prophylaxis (93.9 vs. 97.1%, p=1.00), and heparin was the most commonly used intraoperative thrombosis prophylaxis (84.8 vs. 85.3%, p=1.00) among the subjects of Trusteel® and Ethisteel® groups. Good outcome of surgery was noted in both groups, without any perioperative complication.

Post-operative profile

**Figure 3:**
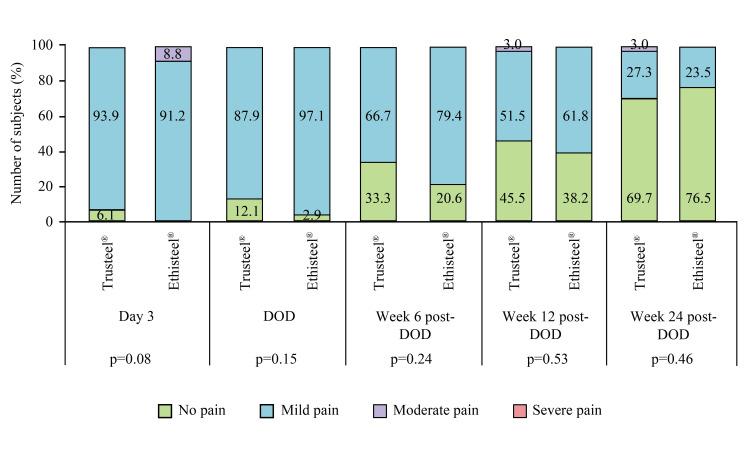
Proportion of subjects with subjectively observed grade of wound pain at rest in Trusteel® (n=33) and Ethisteel® (n=34) groups DOD: Day of discharge Trusteel®: Healthium Medtech Limited, Bengaluru; Ethisteel®: Ethicon, Johnson & Johnson, Cincinnati, USA

No post-operative complications were noted related to superficial and deep sternal wound infection, wire fracture, reoperation, suture-related challenges, and mediastinitis. One subject of the Ethisteel® group died after Week 12 post-DOD follow-up due to an index disease condition; the causality of the event was not related to the study device. However, as the subject was excluded from the PP analysis set, mortality was not marked. Severe cough was only present in 11.8% of subjects of Ethisteel® groups at Day 3 (p=0.50), and moderate cough in both Trusteel® and Ethisteel® groups (9.1 vs. 17.7%) at DOD (p=0.53). Reduced frequency of cough was subjectively observed at post-DOD follow-ups in both groups as found at Week 6 post-DOD (9.1 vs. 20.6%, p=0.19) and Week 12 post-DOD (3.0 vs. 5.9%, p=1.00). In Trusteel® and Ethisteel® groups, mild and moderate pain during coughing on Day 3 was experienced by 33.3 vs. 44.1% and 3.0 vs. 8.8% subjects respectively (p=0.40). At DOD, and Week 6 and 12 post-DOD, 27.3 vs. 38.2% (p=0.98), 9.1 vs. 14.7% (p=0.30), and 3.0 vs. 5.9% (p=1.00) subjects respectively of Trusteel® vs. Ethisteel® group had mild pain during coughing. Mean VAS of subjects who had cough was comparable at Day 3 (22.3±12.3 vs. 27.9±18.3, p=0.19), DOD (17.9±10.1 vs. 21.9±9.9, p=0.24), Week 6 post-DOD (19.3±10.2 vs. 13.3±11.3, p=0.35) and 12 post-DOD (19.0 vs. 23.5±2.1, p=1.00) between Trusteel® vs. Ethisteel® groups. At the last follow-up, none of the subjects had a cough. After recovering from anesthesia, all subjects in the Trusteel® group and 97.1% of subjects in the Ethisteel® group complained of mild pain, while only 2.9% of subjects in the Ethisteel® group had moderate pain (p=0.99). The mean VAS of wound pain was subjectively recorded as 22.2±10.1 in Trusteel® and 23.7±12.5 in the Ethisteel® group, and the result was comparable (p=0.30). The proportion of subjects with subjectively observed grade of wound pain at rest at all post-operative visits was comparable between the groups (Figure 3).

**Figure 4:**
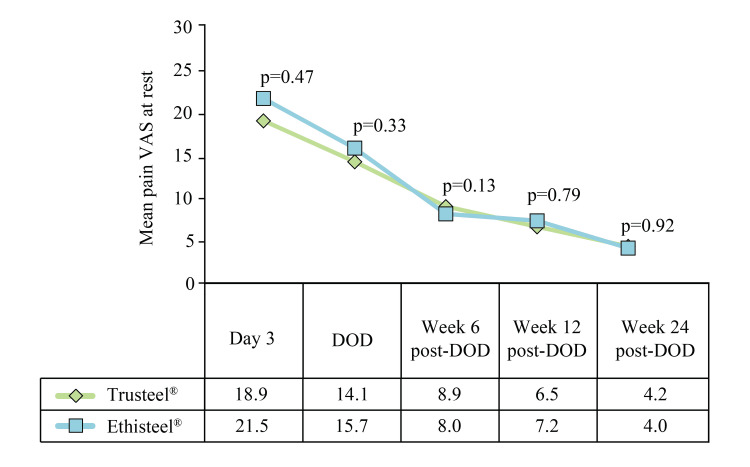
Subjectively observed mean wound pain at rest in Trusteel® (n=33) and Ethisteel® (n=34) groups Mann-Whitney U test was used to compare mean pain VAS scores, DOD: Day of discharge, VAS: Visual analogue scale Trusteel®: Healthium Medtech Limited, Bengaluru; Ethisteel®: Ethicon, Johnson & Johnson, Cincinnati, USA

Also, subjectively observed mean VAS of wound pain at rest at all post-operative visits was comparable between the groups (Figure 4).

**Figure 5:**
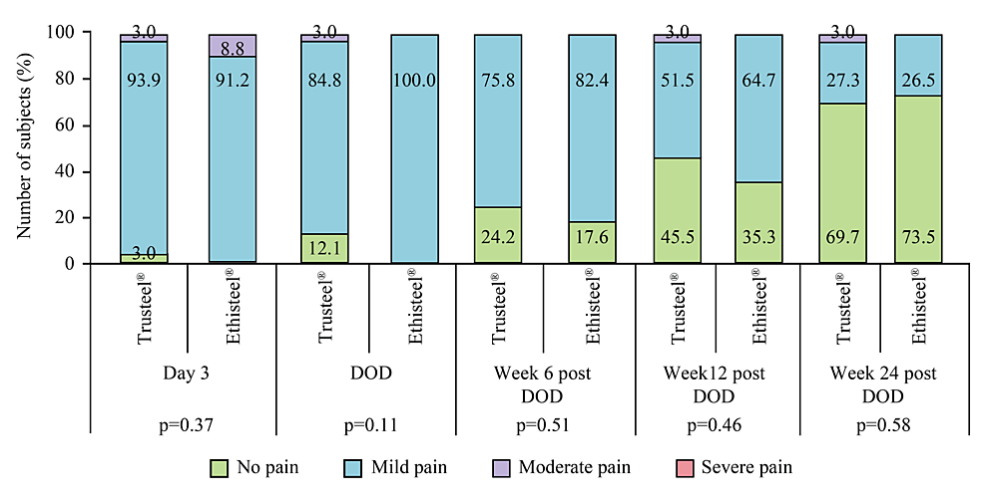
Proportion of subjects with subjectively observed grade of wound pain on movement in Trusteel® (n=33) and Ethisteel® (n=34) groups DOD: Day of discharge Trusteel®: Healthium Medtech Limited, Bengaluru; Ethisteel®: Ethicon, Johnson & Johnson, Cincinnati, USA

The proportion of subjects with subjectively observed grade of wound pain on movement at all post-operative visits was comparable between the groups (Figure 5).

**Figure 6:**
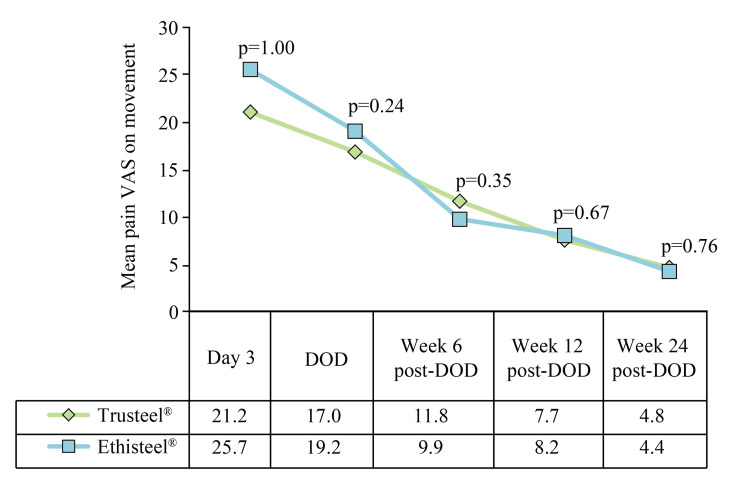
Subjectively observed mean wound pain on movement in Trusteel® (n=33) and Ethisteel® (n=34) groups Mann-Whitney U test was used to compare mean pain VAS scores, DOD: Day of discharge, VAS: Visual analogue scale Trusteel®: Healthium Medtech Limited, Bengaluru; Ethisteel®: Ethicon, Johnson & Johnson, Cincinnati, USA

Also, subjectively observed mean VAS of wound pain on movement at all post-operative visits was comparable between the groups (Figure 6).

**Figure 7:**
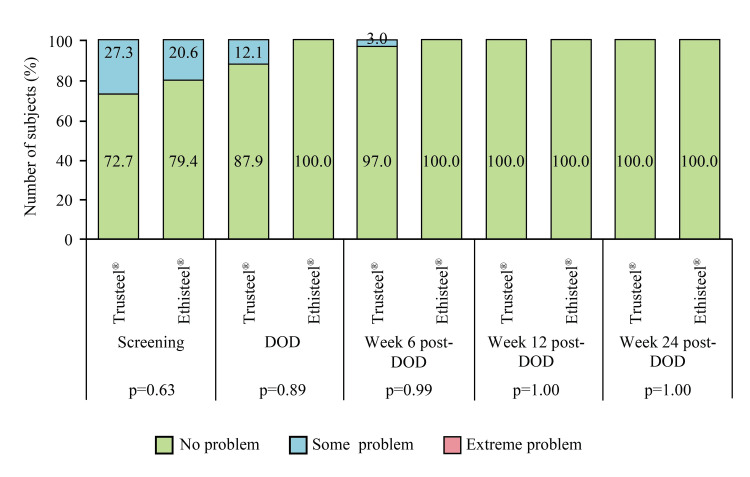
EuroQoL subjective assessment of mobility in Trusteel® (n=33) and Ethisteel® (n=34) groups DOD: Day of discharge. Trusteel®: Healthium Medtech Limited, Bengaluru; Ethisteel®: Ethicon, Johnson & Johnson, Cincinnati, USA

All subjects had clinically stable sternum at all follow-ups. As per the investigator's discretion and institutional protocol, a chest radiograph was performed in both Trusteel® and Ethisteel® groups on Day 3 (24.2 vs. 32.4%, p=0.46), DOD (21.2 vs. 26.5%, p=0.61), Week 6 post-DOD (3.0 vs. 0%, p=0.31) and Week 24 post-DOD (3.0 vs. 0%, p=0.31). Among all of them, a mid-sternum line of lucency of ≤2 mm was present. However, none of the subjects had a displacement of any sternum wire or any obvious interruption or dislocation. No readmission was observed, and durations of ICU and hospital stay were comparable between the groups (Table 2). In addition, the return to day-to-day activity as well as work was comparable between the groups (Table 2). Global assessment of EQ-5D showed improvement in the mobility of subjects in both Trusteel® and Ethisteel® groups (Figure 7).

**Figure 8:**
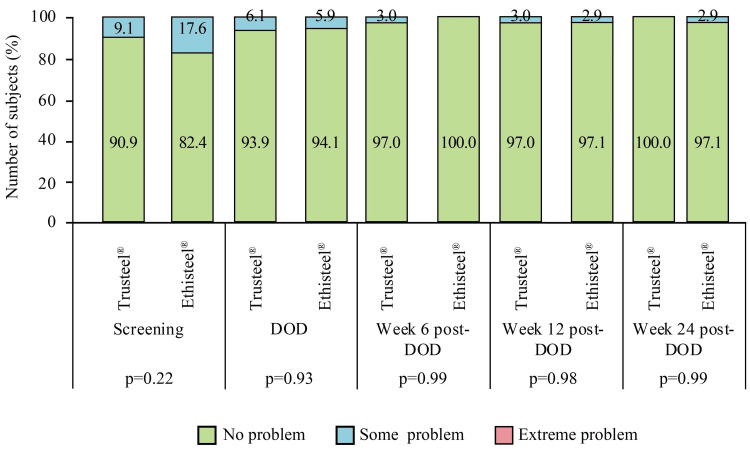
EuroQoL subjective assessment of self-care in Trusteel® (n=33) and Ethisteel® (n=34) groups DOD: Day of discharge. Trusteel®: Healthium Medtech Limited, Bengaluru; Ethisteel®: Ethicon, Johnson & Johnson, Cincinnati, USA

Moreover, an improvement in taking self-care at week 24 was reported by the subjects in both groups (Figure 8).

**Figure 9:**
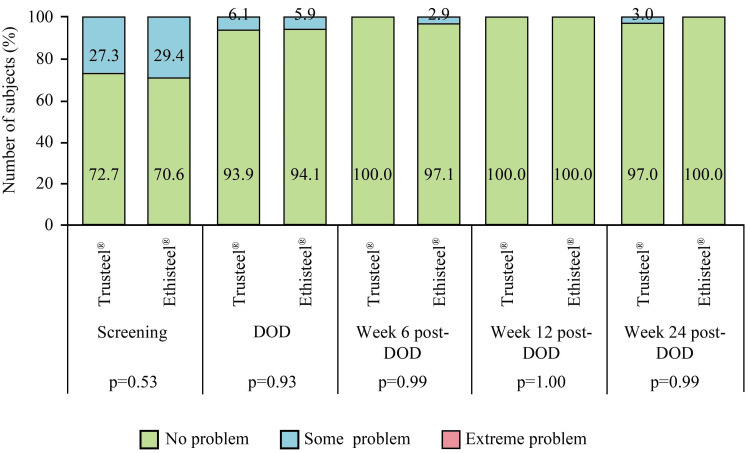
EuroQoL subjective assessment of usual activities in Trusteel® (n=33) and Ethisteel® (n=34) groups DOD: Day of discharge. Trusteel®: Healthium Medtech Limited, Bengaluru; Ethisteel®: Ethicon, Johnson & Johnson, Cincinnati, USA

With each subsequent follow-up, an improvement was observed in the subjects’ usual activities (Figure 9).

**Figure 10:**
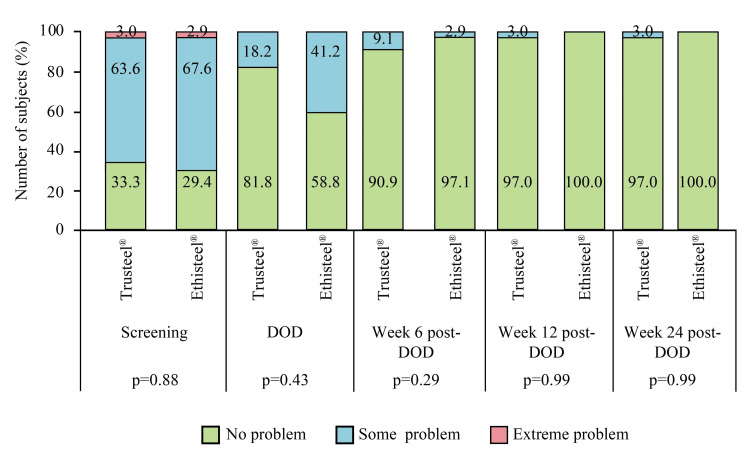
EuroQoL subjective assessment of pain/discomfort in Trusteel® (n=33) and Ethisteel® (n=34) groups DOD: Day of discharge. Trusteel®: Healthium Medtech Limited, Bengaluru; Ethisteel®: Ethicon, Johnson & Johnson, Cincinnati, USA

No pain or discomfort was reported by the majority of subjects of both groups at week 24 (Figure 10).

**Figure 11:**
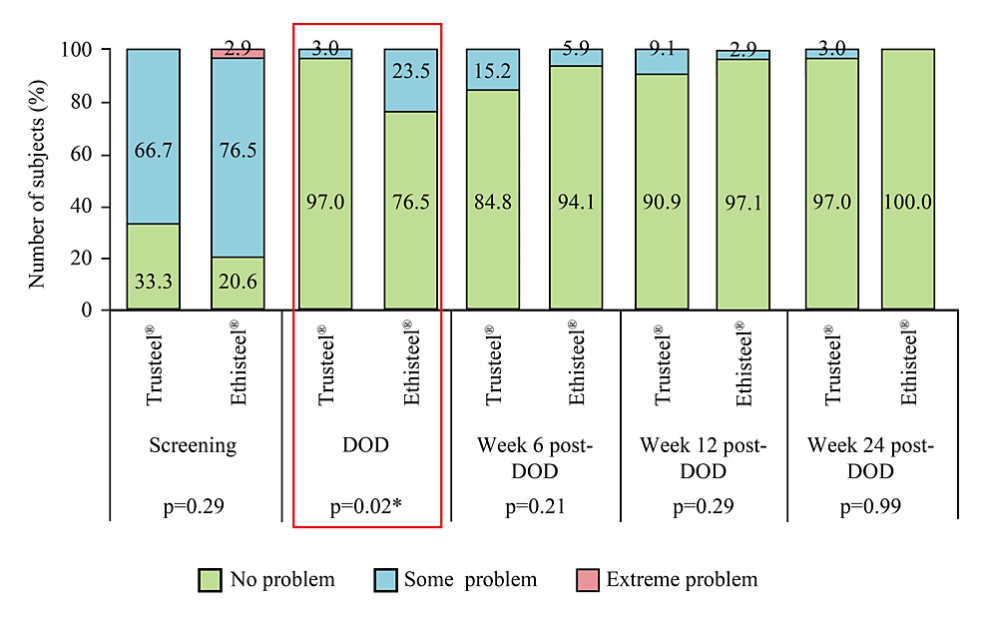
EuroQoL subjective assessment of anxiety/depression in Trusteel® (n=33) and Ethisteel® (n=34) groups DOD: Day of discharge. *p<0.05 Trusteel®: Healthium Medtech Limited, Bengaluru; Ethisteel®: Ethicon, Johnson & Johnson, Cincinnati, USA

An improvement in anxiety or depression was also recorded with each passing follow-up (Figure 11).

**Figure 12:**
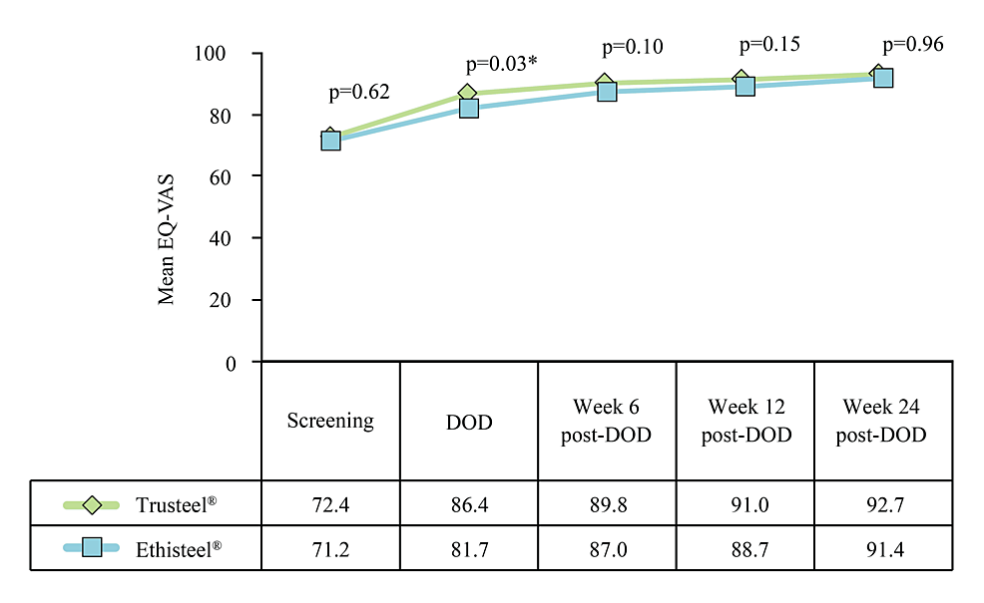
EQ-VAS assessment in Trusteel® (n=33) and Ethisteel® (n=34) groups t-test was used to compare mean EQ-VAS score. DOD: Day of discharge. Trusteel®: Healthium Medtech Limited, Bengaluru; Ethisteel®: Ethicon, Johnson & Johnson, Cincinnati, USA

Furthermore, both groups exhibited a similar improvement in the subjects' quality of life, as evidenced by EQ-VAS (Figure 12).

Mild AEs were recorded in this study, the incidence of which was not related to the study devices. In the Trusteel® group, general body pains (6.1%), and in the Ethisteel® group, distension of stomach and constipation (5.9%), general body pains (5.9%), abdominal pain (2.9%), serous discharge (2.9%), drowsiness and frequent urination (2.9%), and mild pain at the right side of chest (2.9%) were observed.

## Discussion

Sternotomy is frequently performed for conductance of cardiac surgical procedures [6]. Despite its continuous use, surgeons are still searching for new closure methods to combat several sternotomy-related complications such as sternal instability, sternal dehiscence, mediastinitis, and infection [9]. The complications ultimately result in high morbidity and mortality, increase financial burden [2,10], and jeopardize patient recovery [6]. The lower sternum being the most unstable form requires additional reinforcement for stability; a cadaveric experiment has recommended reinforcement with stainless steel coils that strengthens sternal stability [11]. The present study compared Trusteel® and Ethisteel® surgical steel sutures with respect to the incidence of sternal dehiscence within 26 weeks of median sternotomy closure. Existing evidence indicated sternal dehiscence as one of the major complications arising in median sternotomy. A retrospective study on median sternotomy in 14,171 people reported 298 incidents of sternal dehiscence [12]. Similarly, a prospective study from China on 689 people showed 11-14 cases of sternotomy-related complications, comprising sternal dehiscence, post-operative bleeding, and deep sternal wound infection [13]. Contrary to these findings, the present study found no incidence of post-operative sternal dehiscence.

The Centers for Disease Control and Prevention guidelines state that 1-3% of patients develop deep sternal wound infection after median sternotomy [14]. A prospective multi-institutional cohort study on 5158 patients reported increased hospitalization, readmissions, and mortality related to post-surgery mediastinal infection than non-mediastinal infected subjects [15]. Listewnik et al. reported that the duration of hospital stay doubles with sternal dehiscence [12]. A retrospective study on post-sternotomy dehiscence or mediastinitis revealed the failure of first revision with the requirement of reoperation in 9% of subjects; several risk factors like older age, obesity, lower body mass index, hypertension, diabetes, chronic renal failure, and smoking history during hospitalization further increased the risk of mortality by 19% [16]. The Society of Thoracic Surgeons Practice Guideline has recommended proper sternal closure to provide stability and reduce the risk of infection, specifically in high-risk patients [17]. In the United States, the Centers for Medicare & Medicaid Services have applied several measures to penalize hospitals for the occurrence of mediastinitis, putting healthcare providers under pressure to control deep sternal wound infection [10]. Patient outcomes and healthcare optimization following median sternotomy is an unmet goal that enforced the Spanish Society of Cardiovascular Infections, the Spanish Society of Thoracic and Cardiovascular Surgery, and the Biomedical Research Centre Network for Respiratory Diseases to publish a consensus evidence-based document for the management of post-surgical mediastinitis. The recommendations include stable wound closure and the use of surgical steel wires for sternum closure [18]. The present study witnessed no incidence of mediastinitis, superficial and deep sternal wound infection, and requirement for reoperation after sternal closure with Trusteel® or Ethisteel® steel suture in subjects undergoing sternotomy. As a result, no prolonged hospital stays, or readmission was recorded in either group.

Coughing after sternotomy puts extra stress on the sternum through lateral displacement and transverse shear [19]. Violent coughing following sternal closure may result in sternal instability and serosanguineous discharge from the wound that may require to be reoperated [20]. In the present study, not too frequent coughing was recorded, the intensity of which was decreased with each follow-up, and at the last visit no subject had cough. Additionally, all subjects had clinically stable sternum without any incidence of sternum wire displacement, wire fracture, or any obvious interruption/dislocation. Chronic pain is another major health concern affecting both the quality of life and the subject productivity [21]. Chronic post-sternotomy pain manifests numbness, palpation-associated severe tenderness, and allodynia accompanied by sustained pain along the anterior chest wall that may remain at least for three months [22] or longer [22,23]. Initially, all subjects of the present study had pain that gradually reduced and toward the end of the study majority had no pain during rest or movement.

Evaluation of EQ-5D-3L provides knowledge of recovery and post-surgery physical functioning [24] and has marked responsiveness among patients with coronary heart disease [25]. A recent study by Akowuah et al. found that the subjects recovered within 12 weeks of conventional median sternotomy and returned to regular physical activities [26]. Subjects of both Trusteel® and Ethisteel® groups returned to normal day-to-day activities by ~10 days without any complications. In addition, at the last follow-up, the majority of subjects reported no problems related to mobility, self-care, usual activities, pain/discomfort, and anxiety/depression, and mostly graded their health as the best imaginable health. The AEs of this study were not related to the suture material and did not intervene with the study outcomes.

Limitations and strengths of the study

The present study is a single-blind study where the surgeons/practitioners/enquiring staff could not be blinded and this is a limitation of the study, as there are chances of potential bias in the overall assessment of the intraoperative suture handling parameters. Though the use of a suitable technique and methodological approach led to no incidence of sternal dehiscence among the subjects of both groups, it resulted in similar outcomes between the groups. However, the current update on the standard of care in sternal closure following surgical procedures by median sternotomy is provided from an Indian perspective. This is the strength of the study. In addition, the study findings manifested and validated the clinical use of Trusteel® in a larger population.

## Conclusions

Sternal closure following open surgical procedures on the heart with Trusteel® or Ethisteel® suture resulted in no incidence of post-sternotomy sternal dehiscence within 26 weeks of index surgery. Additionally, comparable findings of intraoperative handling of suture (except stretch capacity), mortality and other complications, operative time, ICU/hospital stay, return to normal day-to-day activities and work, adverse events, and subject satisfaction and general well-being indicated clinical equivalence of the Trusteel® surgical steel suture to Ethisteel® surgical steel suture. Therefore, closure of the sternum using Trusteel® or Ethisteel® surgical steel sutures in subjects undergoing surgical procedures by median sternotomy is safe and effective.
